# Supplementary material for: DMRforPairs: identifying Differentially Methylated Regions between unique samples using array based methylation profiles
Source: BMC Bioinformatics. 2014 May 15;15:141. doi: 10.1186/1471-2105-15-141 (PMC4046028; doi:10.1186/1471-2105-15-141)
Supplement: Additional file 2 — DMRforPairs output for the comparison of A431-MCF7 and NA17018-NA17105. Please start from the HTML files in each folder. Available via the BMC Bioinformatics website. [file 1471-2105-15-141-S2.zip › 1394847754114233_MOESM2_ESM/A431_MCF7/figures/2144.pdf]

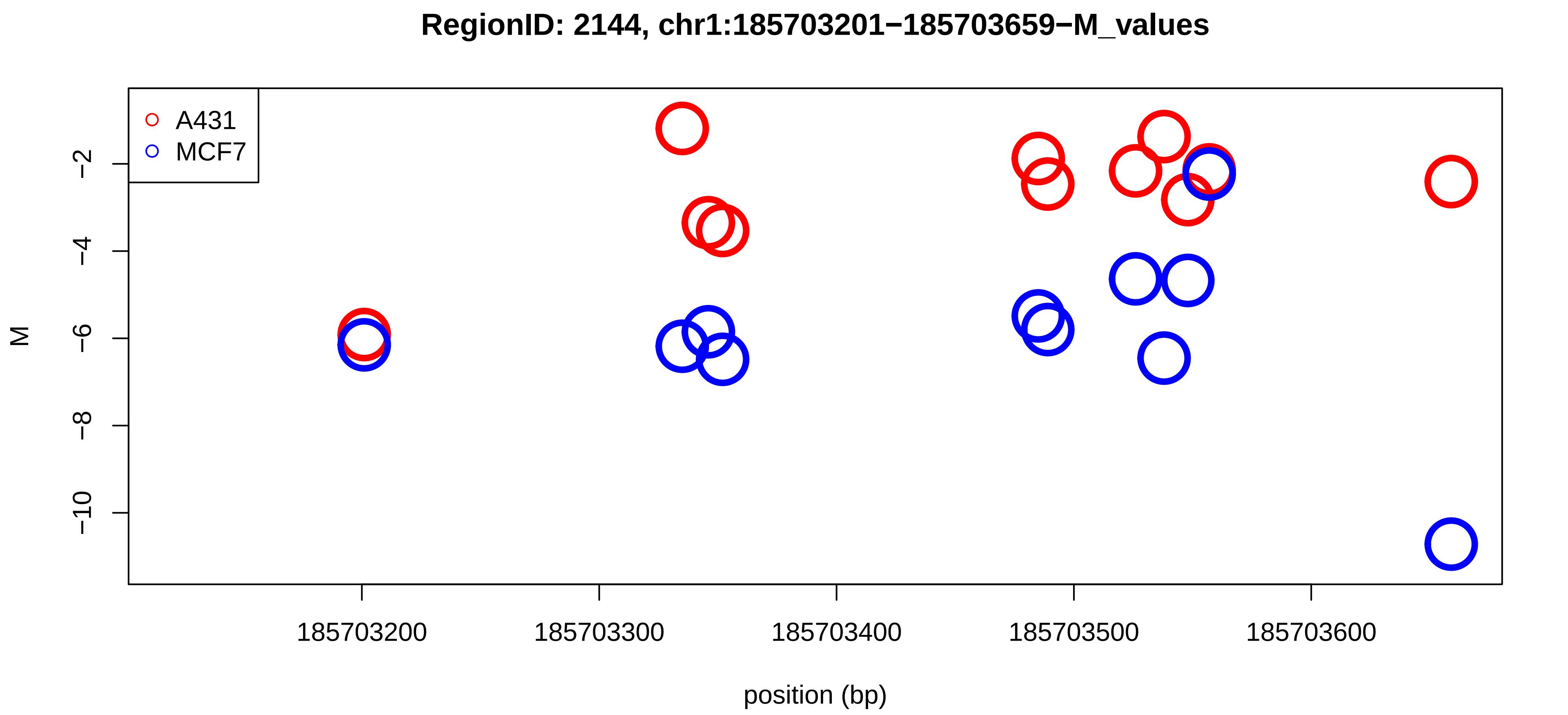
RegionID: 2144, chr1:185703201−185703659−M_values
A431
MCF7
M
−2
−4
−6
−8
−10
185703200
185703300
185703400
185703500
185703600
position (bp)

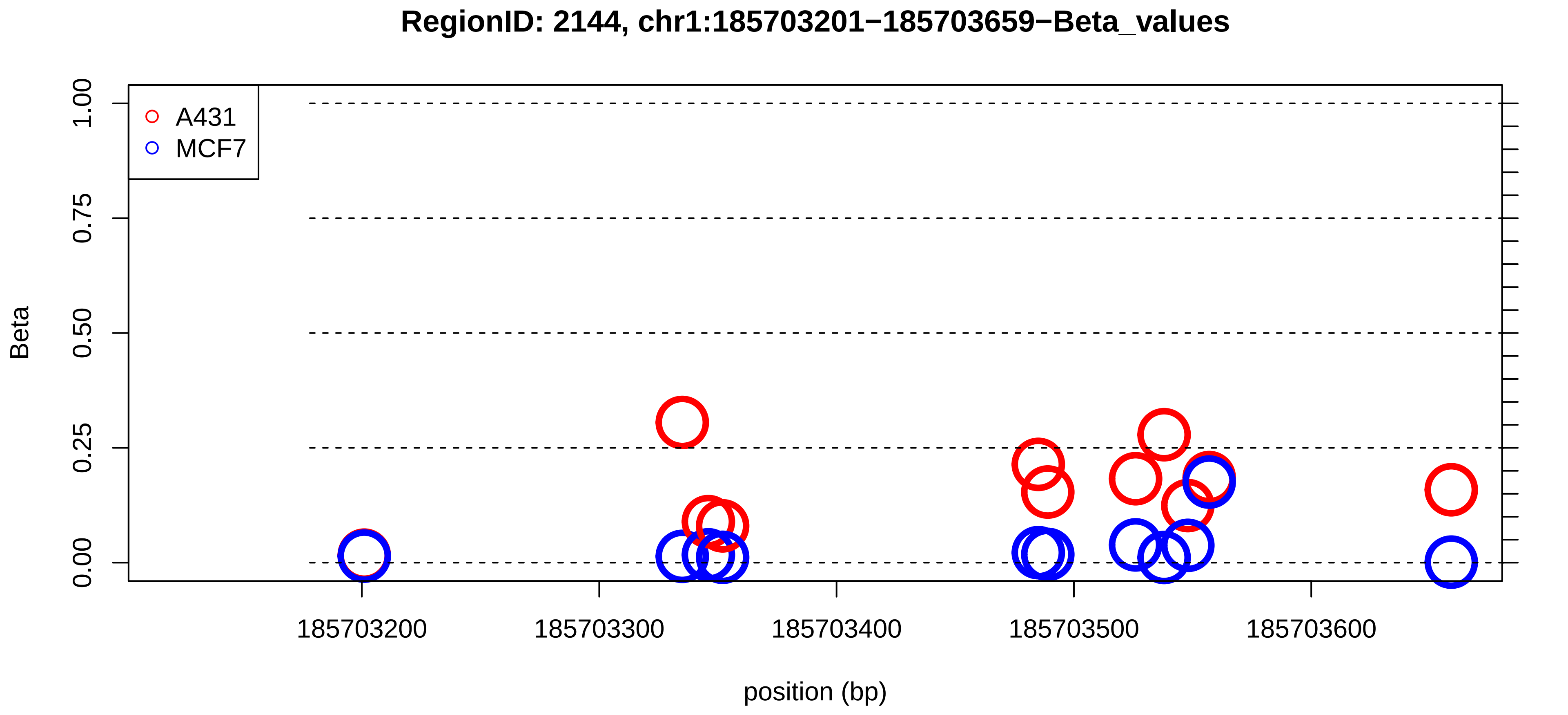
RegionID: 2144, chr1:185703201−185703659−Beta_values
A431
MCF7
Beta
0.00
0.25
0.50
0.75
1.00
185703200
185703300
185703400
185703500
185703600
position (bp)

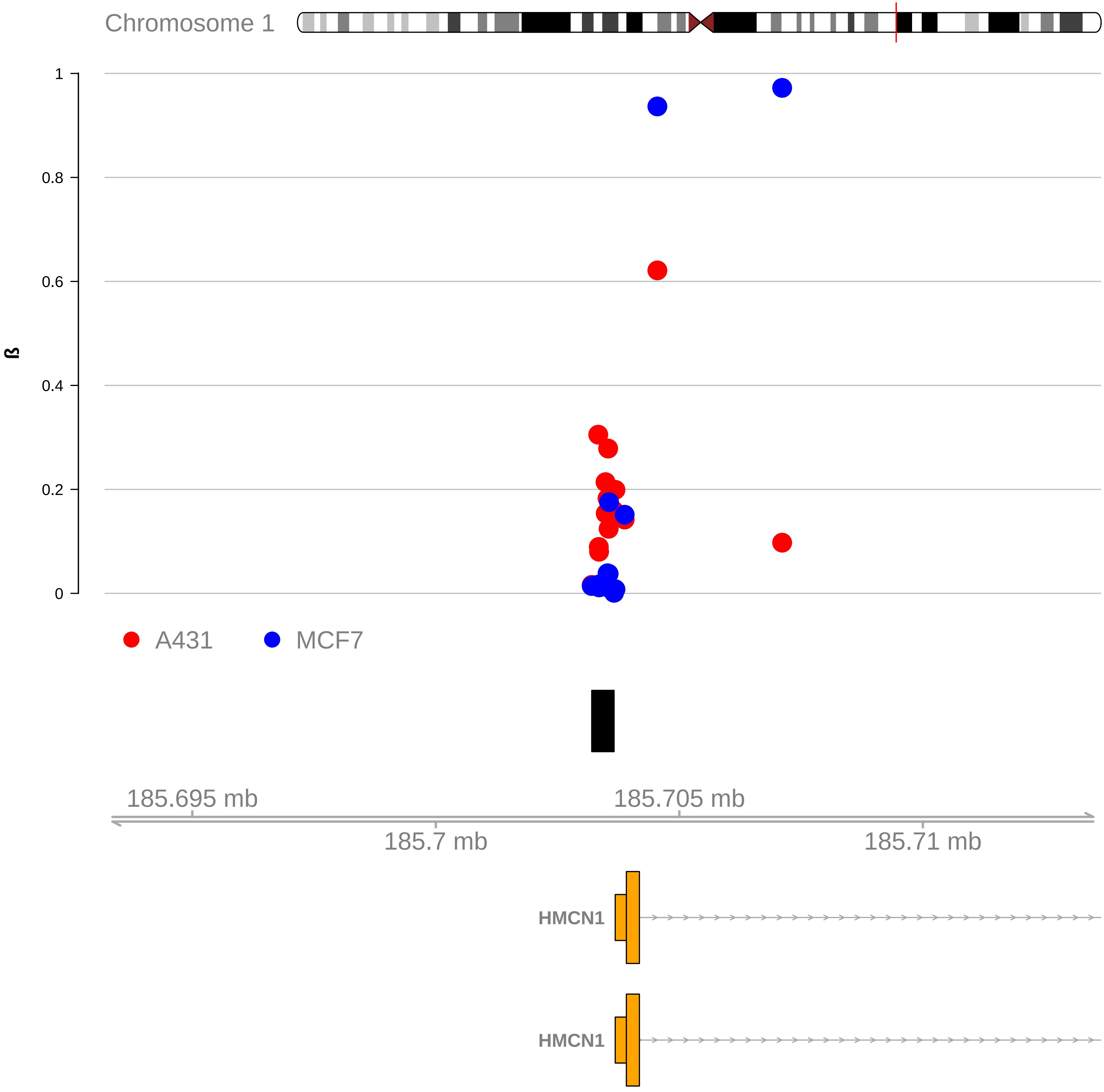
Chromosome 1
1
0.8
0.6
0.4
0.2
0
ß
A431
MCF7
185.695 mb
185.705 mb
185.7 mb
185.71 mb
HMCN1
HMCN1
